# Meta-analysis of *GABRB2* polymorphisms and the risk of schizophrenia combined with GWAS data of the Han Chinese population and psychiatric genomics consortium

**DOI:** 10.1371/journal.pone.0198690

**Published:** 2018-06-12

**Authors:** Tian Zhang, Jun Li, Hao Yu, Yongyong Shi, Zhiqiang Li, Linyan Wang, Ziqi Wang, Tianlan Lu, Lifang Wang, Weihua Yue, Dai Zhang

**Affiliations:** 1 Peking University Sixth Hospital, Beijing, China; 2 Peking University Institute of Mental Health, Beijing, China; 3 Key Laboratory of Mental Health, Ministry of Health (Peking University), Beijing, China; 4 National Clinical Research Center for Mental Disorders, (Peking University Sixth Hospital), Beijing, China; 5 Department of Psychiatry, Jining Medical University, Jining, Shandong, China; 6 Affiliated Hospital of Qingdao University and Biomedical Sciences Institute of Qingdao University (Qingdao Branch of SJTU Bio-X Institutes), Qingdao University, Qingdao, China; 7 Bio-X Institutes, Key Laboratory for the Genetics of Developmental and Neuropsychiatric Disorders (Ministry of Education), Collaborative Innovation Center for Brain Science, Shanghai Jiao Tong University, Shanghai, China; 8 Institute of Social Cognitive and Behavioral Sciences, Shanghai Jiao Tong University, Shanghai, China; 9 Institute of Neuropsychiatric Science and Systems Biological Medicine, Shanghai Jiao Tong University, Shanghai, China; 10 Department of Psychiatry, First Teaching Hospital of Xinjiang Medical University, Urumqi, China; 11 Changning Mental Health Center, Shanghai, China; 12 Peking-Tsinghua Center for Life Sciences, Peking University, Beijing, China; 13 PKU-IDG/McGovern Institute for Brain Research, Peking University, Beijing, China; Kunming Institute of Zoology, Chinese Academy of Sciences, CHINA

## Abstract

Schizophrenia (SCZ) is a severe psychiatric disorder with evidence of a strong genetic component in the complex etiologies. Some studies indicated that gamma-aminobutyric acid (GABA)_A_ receptor β2 subunit gene (*GABRB2*) was associated with SCZ. Other studies reported a negative association. Moreover, the results of two previous meta-analyses of *GABRB2* with SCZ were inconsistent and the sample sizes were limited. Therefore, an updated meta-analysis combined with genome-wide association study (GWAS) data of the Han Chinese population and Psychiatric Genomics Consortium (PGC) was performed. Available case–control and family-based genetic data were extracted from association studies, and the GWAS data were included. The findings showed no association between six single-nucleotide polymorphisms of *GABRB2* (rs6556547, rs1816071, rs1816072, rs194072, rs252944, and rs187269) and SCZ in a total of 51,491 patients and 74,667 controls. The ethnic subgroup analysis revealed no significant association in Asian populations. Since the PGC data of SCZ (SCZ-PGC, 2014) contained 3 studies of Asian populations (1866 patients and 3418 controls), only the data of European samples in SCZ-PGC were used for the meta-analysis of the Caucasian population in the present study. The result still showed no association in the Caucasian population. In conclusion, the present meta-analysis on combined data from GWASs of the Han Chinese population and PGC suggested that *GABRB2* polymorphisms might not be associated with SCZ.

## Introduction

Schizophrenia (SCZ) is a complex psychiatric disorder affecting approximately 1% of the global population and manifesting as positive symptoms (delusions and hallucinations), negative symptoms (impaired motivation, unusual speech or behavior, and social withdrawal), and cognitive impairment [1,2]. As SCZ has a high heritability of 70%–85% and a tenfold increase in the risk of siblings of probands, genetic factors are vital in the pathogenesis of SCZ [3,4]. However, the specific role of genetic factors in SCZ remains unclear.

Gamma-aminobutyric acid (GABA) is the main inhibitory neurotransmitter in the brain. Abnormalities of specific cortical inhibitory neurons and GABA may contribute to imbalances in excitatory/inhibitory signaling in the brain and hence GABA is crucial in the pathophysiology of SCZ [5]. GABA exerts its inhibitory activity by binding to two types of receptors (GABA_A_ and GABA_B_ receptors). GABA_A_ receptors are ligand-gated Cl^−^ channels responsible for most of the physiological actions of GABA [6]. The functions of GABA_A_ receptors are associated with psychiatric diseases, such as SCZ [7–11]. Postmortem studies indicated that a decline in the biosynthesis of cortical GABA led to compensatory upregulation of GABA_A_ receptors and downregulation of GABAergic cortical function in SCZ [12]. Among GABA_A_ receptor subunit genes, gene cluster, including *GABRB2*, *GABRA6*, *GABRA1*, and *GABRG2*, attract attention. These genes, which are highly expressed in the brain, are located on chromosome 5q34 [13]. A meta-analysis of genome-wide linkage scans of SCZ identified chromosome 5q23.2–q34 as the second most significant risk locus in the genome [14]. Moreover, a genome-wide association study (GWAS) for SCZ in Japanese population showed association signals on the GABA_A_ receptor subunit gene cluster on chromosome 5q34 [15].

Among the GABA_A_ receptor subunit gene cluster on chromosome 5q34, the GABA_A_ receptor β_2_ subunit gene (*GABRB2*) with SCZ has gained interest. GABRB2 regulates the intracellular Ca^2+^ concentration, which is important for the nervous system [16]. Postmortem samples of SCZ indicated alterations in the expression of GABA receptor β_2_ subunit protein [17]. Furthermore, abnormal N-glycosylation of GABA receptor β_2_ subunit and altered methylation of *GABRB2* were also found in patients with SCZ [18,19].

To date, association studies have been carried out to evaluate the association between *GABRB2* polymorphisms and risk of SCZ. Positive associations between *GABRB2* polymorphisms and SCZ were found in Asian [20–22] and Caucasian populations [21,23]. However, other studies reported no association between *GABRB2* and SCZ in Asian [24,25] or Caucasian population [26]. The results of previous two meta-analyses of *GABRB2* with SCZ were inconsistent [27,28]. Allen *et al*. reported that rs6556547, rs1816071, rs1816072, and rs194072 of *GABRB2* were significantly associated with SCZ in the Caucasian population (about 1863 patients with SCZ and 1631 controls), whereas another study indicated an association of rs1816071 in Caucasian population and rs1816072 in Asian and Caucasian populations with SCZ (about 2240 patients and 2093 controls). Later, other association studies between *GABRB2* and SCZ showed controversial results. Some studies suggested a positive association [29,30], whereas others indicated a negative correlation [31]. Moreover, GWASs were often performed to detect causal or risk-conferring genes for common diseases [32]. The Psychiatric Genomics Consortium (PGC) has reported a few meta-analyses for SCZ [33–35]. However, GWASs on SCZ in non-European populations were limited to small sample sizes. Recently, Yu *et al*. performed a two-stage GWAS on SCZ comprising 4384 patients and 5770 controls, followed by replication in an additional 4339 patients with SCZ and 7043 controls of Chinese Han ancestry [36]. Li *et al*. recruited 7699 SCZ cases and 18,327 controls in Chinese Han and conducted a GWAS for SCZ [37]. Considering the inconsistencies between the previous two meta-analyses and the limited sample size, the present study performed an updated meta-analysis on the combined GWAS data of the SCZ-PGC and Chinese Han population.

Available case–control and family-based genetic data from association studies and the PGC data of SCZ, as well as GWAS data of Han Chinese ancestry, were extracted in this study. An updated meta-analysis was performed to explore the association of six single-nucleotide polymorphisms (SNP) (rs6556547, rs1816071, rs1816072, rs194072, rs252944, and rs187269) in *GABRB2* and the risk of SCZ.

## Material and methods

### Identification and selection of studies

Electronic databases, such as Essential Science Indicators, PubMed, Embase, and China National Knowledge Infrastructure were searched for all relevant reports (the last search update was on December 31, 2017) using the search following terms: “*GABRB2*” OR “GABA(A) receptor subunit beta2 gene” AND “schizophrenia”. The search was limited to peer-reviewed, published studies. Studies in English language only were considered. As a complementary measure, references of correlative studies were retrieved to search for other eligible studies. The inclusion criteria were as follows: (1) studies involving the associations between *GABRB2* and SCZ (2) those using a case–control or family-based design and (3) those providing complete data on allele frequencies or genotype frequencies of patients and controls. The exclusion criteria were as follows: (1) review studies, animal studies, simply commentaries, case reports, or unpublished reports (2) studies not reporting the complete data and (3) duplicate publications.

### Meta-analysis sample

Nine eligible candidate association studies were finally adopted with a sample size of 3434 patients and 3232 controls as well as 499 trios. These studies comprised seven case–control studies, one family-based study, and one study with both case–control and family-based designs. Based on the ancestry, four Asian association studies and four Caucasian association studies, as well as one study performed on both Asian and Caucasian population, were included. Moreover, data from the SCZ-PGC GWAS (SCZ-PGC, 2014) and two GWASs for SCZ in the Han Chinese population were used for the meta-analysis. The data of the PGC GWAS (SCZ-PGC, 2014) were downloaded from the PGC website (http://www.med.unc.edu/pgc; SCZ2). The current SCZ-PGC GWAS consisted of 49 case-control samples (46 of European and 3 of East Asian ancestry, involving 34,241 patients and 45,604 controls) and 3 family-based samples of European ancestry (1235 parent affected-offspring trios). The sample of PGC included 35,476 patients and 46,839 controls. The 2 SCZ GWASs of Chinese Han population comprised 4384 patients and 5770 controls, and 7699 patients and 18,327 controls, respectively. In this meta-analysis, the total samples included 51,491 patients and 74,667 controls.

### Data extraction

Two independent researchers extracted the following information in each study: journal, name of the first author, year of publication, ethnicity, design of the study, diagnostic criteria for SCZ, sample size of patients and controls or family trios, and genotype data or transmission/disequilibrium test (TDT) data. If the data were incomplete, the study authors were requested to provide the missing data.

### Statistical methods

The meta-analysis was performed using the Stata version 14.0 software (Stat Corp., TX, USA). In case–control studies, if allele frequencies or genotype data were incomplete, they were calculated from the existing corresponding information. The allelic data were used to calculate the natural logarithms of odds ratios [Ln (ORs)] and standard errors (SEs). For family-based studies, the Ln (ORs) and SEs of each study were calculated based on the TDT data [38,39]. For the meta-analysis samples, the data of Ln (ORs) and SEs were directly extracted from the GWAS results. The pooled *P* values, ORs, and 95% confidence intervals (CIs) were calculated to investigate the association between the risk of SCZ and *GABRB2* polymorphisms between two population subgroups (Asian and Caucasian). The heterogeneity between studies was tested using the Q-statistic (cutoff: *P* < 0.1), and its magnitude was evaluated using the *I*^*2*^ statistic. The fixed-effects or random-effects models were used according to the heterogeneities (*I*^2^ < 50%, fixed-effects models; *I*^2^ > 50%, random-effects models). Subgroup analysis was conducted by ethnicity (Asian and Caucasian).

Meta-regression was used to investigate the effects of the sample size, study design, and year of publication. The *P* value of meta-regression less than 0.05 was considered to have a significant effect. A sensitivity analysis was also applied to evaluate the effect of each study on the combined ORs by omitting each study in each turn. Publication bias was assessed using funnel plots and Egger’s test. A *P* value less than 0.05 in the Egger’s test indicated significance.

## Results

### Study characteristics

**Fig 1:**
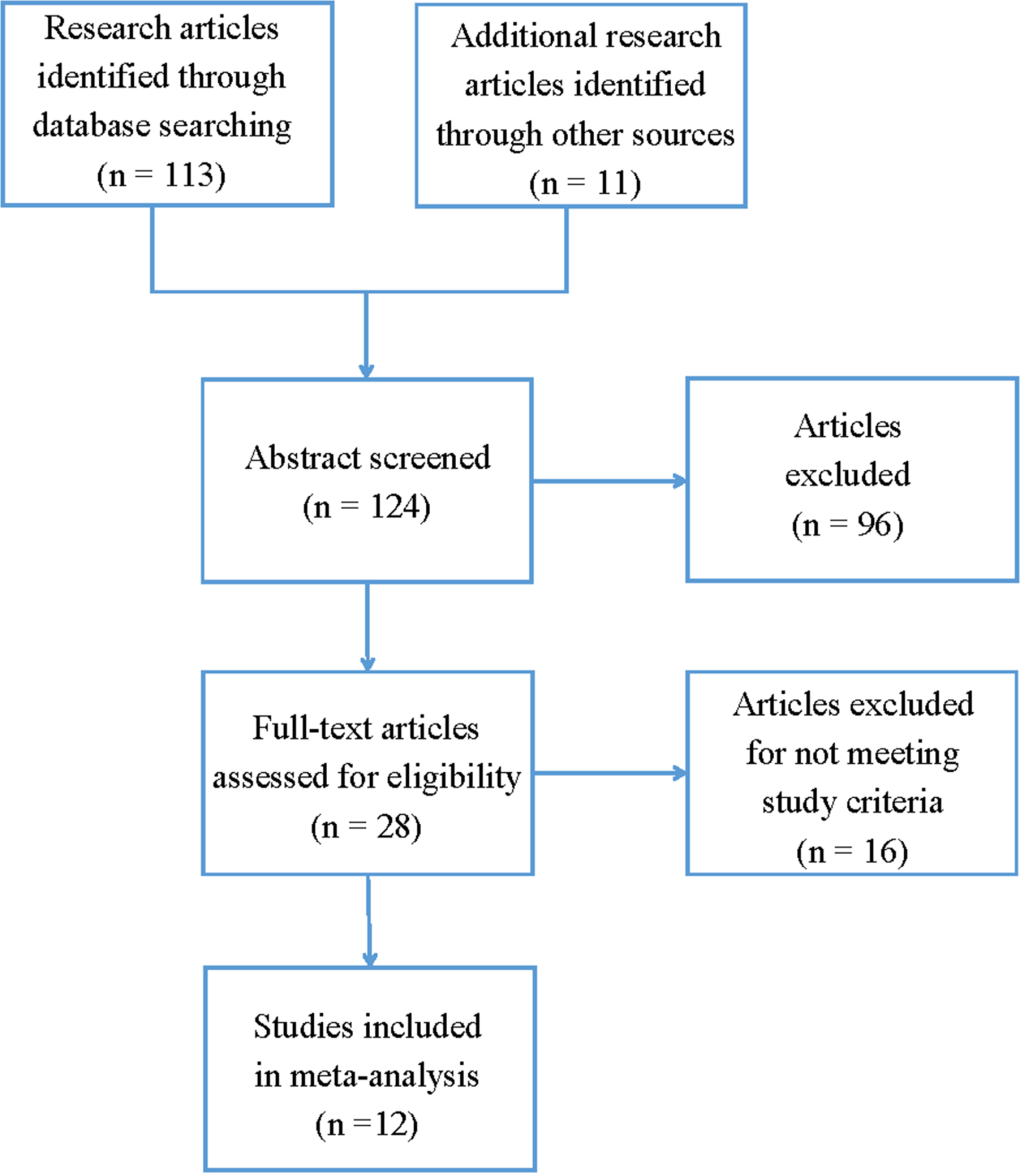
Overview of the literature search and article screening procedure.

The literature search and article screening procedure are shown in Fig 1. Applying the article screening strategy, 28 published articles that might meet the inclusion criteria were identified. After reading full-text studies, 16 studies were excluded, including 2 review studies, 2 animal studies, 3 studies that had overlap samples with other studies, 1 study with no complete data, 7 studies that did not explore the polymorphisms, and 1 study that did not investigate the polymorphisms included in the present meta-analysis. Therefore, the present meta-analysis included data from 12 publications that consisted data from case–control designed studies (3434 patients and 3232 controls), family-based designed studies (499 trios), and 3 GWAS data (SCZ-PGC 2014, 35,476 patients and 46,839 controls; Yu *et al*. 2017, 4383 patients and 5770 controls; Li *et al*. 2017, 7699 patients and 18,327 controls). The observed genotype distribution in the controls was consistent with Hardy–Weinberg equilibrium. The detailed characteristics of each study are described in Table 1.

**Table 1:** Descriptive characteristics of selected studies in meta-analysis.

No.	Author	Year	Sample Size (Case/Control, trios)	Ancestry	Criteria
1	Ikeda *et al*.	2005	288/288	Asian, Japanese	DSM-IV
2	Petryshen *et al*.	2005	321/242	Caucasians, Portuguese	DSM-IV
			111 trios	Caucasians, Portuguese	DSM-IV
			238 trios	Caucasians, German	DSM-IV
3	Zhao *et al*.	2006	31/31	Caucasians, US population	DSM-IV
4	Lo *et al*.	2007	304/207	Asian, Japanese	DSM-IV
			301/190	Caucasians, German	DSM-IV
5	Zhao *et al*.	2007	292/286	Asian, Chinese Han	DSM-III-R
6	Jamra *et al*.	2007	367/360	Caucasians, German	DSM-IV
7	Pun *et al*.	2011	150 trios	Caucasians, US population	DSM-IV
8	Tsang *et al*.	2013	115/117	Asian, Chinese Han	DSM-IV
9	SCZ-PGC	2014	35476/46839	Caucasians and Asian	/
10	Yu *et al*.	2017	4383/5770	Asian, Chinese Han	DSM-IV
11	Balan *et al*.	2017	1415/1511	Asian, Japanese	DSM-IV
12	Li *et al*.	2017	7699/18327	Asian, Chinese Han	DSM-IV

Abbreviations: DSM, Diagnostic and Statistical Manual of Mental Disorders.

### Meta-analysis

The SNPs were selected according to the following criteria: (1) SNPs in *GABRB2* included in most of the related association studies and (2) previously reported positive SNPs in *GABRB2* related to SCZ. Therefore, six SNPs of *GABRB2* (rs6556547, rs1816071, rs1816072, rs194072, rs252944, and rs187269) were selected to perform the present meta-analysis with SCZ. Before combining with GWAS data, all six SNPs in *GABRB2* showed no significant association with SCZ (rs6556547: OR = 1.02 95% CI = 0.88–1.18 *P* = 0.837, rs1816071: OR = 0.97 95% CI = 0.89–1.07 *P* = 0.553, rs1816072: OR = 1.02 95% CI = 0.90–1.16 *P* = 0.731, rs194072: OR = 1.03 95% CI = 0.91–1.16 *P* = 0.673, rs252944: OR = 1.01 95% CI = 0.90–1.15 *P* = 0.829, rs187269: OR = 1.08 95% CI = 0.90–1.29 *P* = 0.398; S1 Table and S1 Fig). Considering the difference in ethnicity, the data for Asian and Caucasian populations were analyzed separately. One SNP rs1816072 (C/T) was significantly associated with SCZ in Asian (OR = 1.18; 95% CI, 1.01–1.39; *P* = 0.043) and Caucasian populations (OR = 0.88; 95% CI, 0.78–0.99; *P* = 0.038), but not in the overall population (S1C Fig).

**Fig 2:**
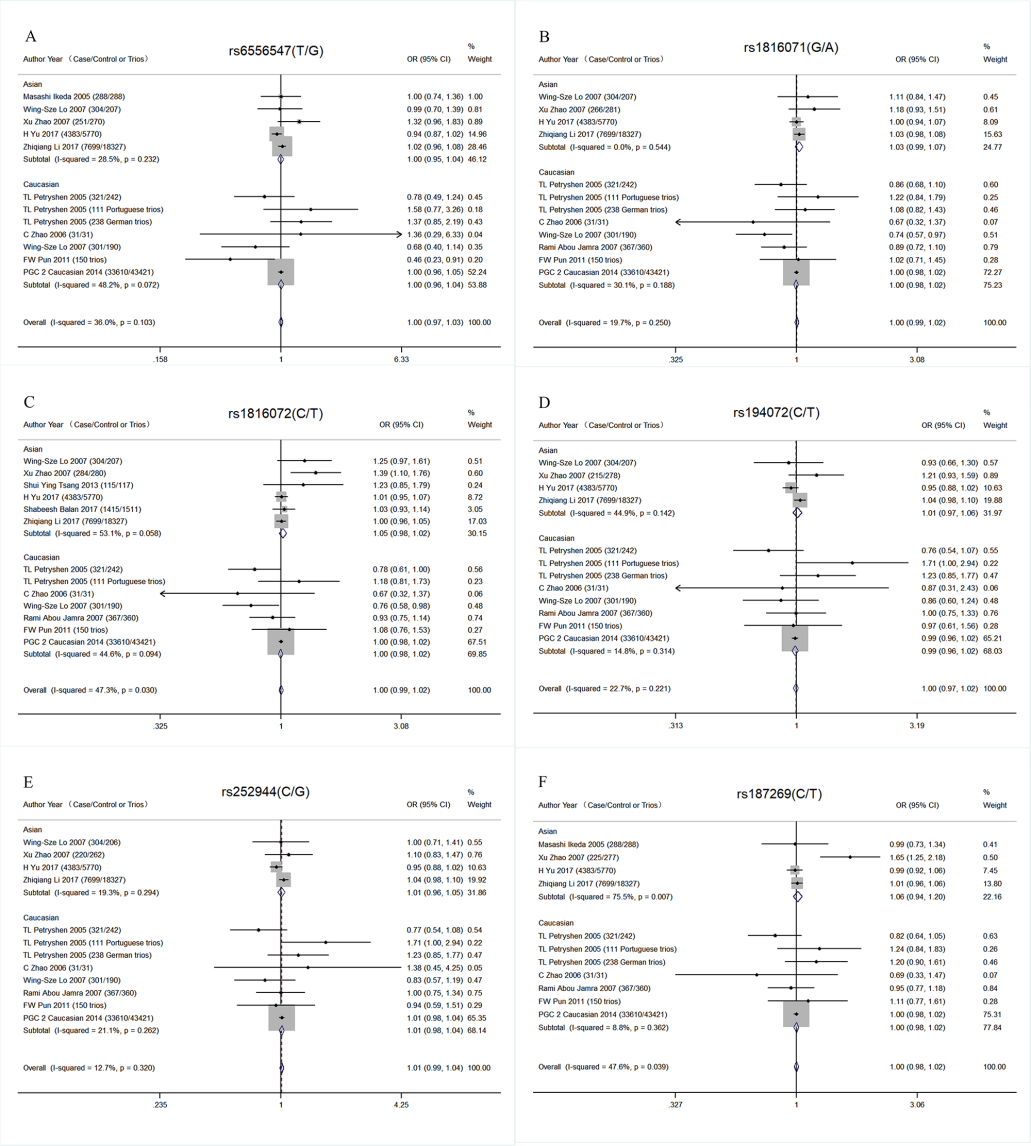
Forest plot of SNPs in *GABRB2* in different ethnic subgroups combined with GWAS schizophrenia data. (A) Meta-analysis of *GABRB2* rs6556547. (B) Meta-analysis of *GABRB2* rs1816071. (C) Meta-analysis of *GABRB2* rs1816072. (D) Meta-analysis of *GABRB2* rs194072. (E) Meta-analysis of *GABRB2* rs252944. (F) Meta-analysis of *GABRB2* rs187269.

Furthermore, three GWAS data were added for meta-analysis. No association was detected in the Asian population (rs6556547: OR = 1.00 95% CI = 0.95–1.04 *P* = 0.913, rs1816071: OR = 1.03 95% CI = 0.99–1.07 *P* = 0.1,93, rs1816072: OR = 1.05 95% CI = 0.98–1.12 *P* = 0.141, rs194072: OR = 1.01 95% CI = 0.97–1.06 *P* = 0.684, rs252944: OR = 1.01 95% CI = 0.96–1.05 *P* = 0.746, rs187269: OR = 1.06 95% CI = 0.94–1.20 *P* = 0.321; Table 2 and Fig 2). The PGC data of SCZ (SCZ-PGC, 2014) predominantly focused on people of European descent. However, it contained 3 studies of Asian population, approximately only 3.5% people of Asian descent (1866 patients and 3418 controls). Only the data of European samples of SCZ-PGC were used for the present meta-analysis in the Caucasian population to decrease the ethnic heterogeneity. The results showed no significant association between *GABRB2* and SCZ (rs6556547: OR = 1.00 95% CI = 0.96–1.04 *P* = 0.968, rs1816071: OR = 1.00 95% CI = 0.98–1.02 *P* = 0.817, rs1816072: OR = 1.00 95% CI = 0.98–1.02 *P* = 0.717, rs194072: OR = 0.99 95% CI = 0.96–1.02 *P* = 0.464, rs252944: OR = 1.01 95% CI = 0.98–1.04 *P* = 0.485, rs187269: OR = 1.00 95% CI = 0.98–1.02 *P* = 0.903; Table 2 and Fig 2). Then, the SCZ-PGC data were applied in the analysis of overall populations (51,491 patients and 74,667 controls). The results were still negative (rs6556547: OR = 1.00 95% CI = 0.97–1.03 *P* = 0.964, rs1816071: OR = 1.00 95% CI = 0.99–1.02 *P* = 0.655, rs1816072: OR = 1.00 95% CI = 0.99–1.02 *P* = 0.756, rs194072: OR = 1.00 95% CI = 0.97–1.02 *P* = 0.708, rs252944: OR = 1.01 95% CI = 0.99–1.04 *P* = 0.448, rs187269: OR = 1.00 95% CI = 0.98–1.02 *P* = 0.887; S2 Table).

**Table 2:** Meta-analytic results of SNPs in *GABRB2* and schizophrenia in different ethnic subgroups combined with GWAS data.

SNP	Population	OR	CI (95%)	*Z*	*P*	Heterogeneity		
						Q	*P*	I-squared
rs6556547	Asian	1.00	0.95–1.04	0.11	0.913	5.59	0.232	28.5%
	Caucasian	1.00	0.96–1.04	0.04	0.968	11.58	0.072	48.20%
	overall	1.00	0.97–1.03	0.04	0.964	17.18	0.103	36.0%
rs1816071	Asian	1.03	0.99–1.07	1.30	0.193	2.14	0.544	0.0%
	Caucasian	1.00	0.98–1.02	0.23	0.817	10.01	0.188	30.10%
	overall	1.00	0.99–1.02	0.45	0.655	13.69	0.250	19.7%
rs1816072	Asian	1.05	0.98–1.12	1.47	0.141	10.67	0.058	53.1%
	Caucasian	1.00	0.98–1.02	0.36	0.717	10.82	0.094	44.60%
	overall	1.00	0.99–1.02	0.31	0.756	22.78	0.030	47.30%
rs194072	Asian	1.01	0.97–1.06	0.41	0.684	5.44	0.142	44.9%
	Caucasian	0.99	0.96–1.02	0.73	0.464	8.21	0.314	14.80%
	overall	1.00	0.97–1.02	0.37	0.708	14.22	0.221	22.7%
rs252944	Asian	1.01	0.96–1.05	0.32	0.746	3.72	0.294	19.3%
	Caucasian	1.01	0.98–1.04	0.70	0.485	8.87	0.262	21.10%
	overall	1.01	0.99–1.04	0.76	0.448	12.60	0.320	12.70%
rs187269	Asian	1.06	0.94–1.20	0.99	0.321	12.24	0.007	75.5%
	Caucasian	1.00	0.98–1.02	0.12	0.903	6.58	0.362	8.80%
	overall	1.00	0.98–1.02	0.14	0.887	19.10	0.039	47.6%

Abbreviations: OR, odds ratio; CI, confidence intervals.

### Meta-regression

Meta-regression was used to investigate the effects of the possible modifiers. It revealed no significant moderation by year of publication, sample size, or study design (S3 Table).

### Sensitivity analysis

Sensitivity analysis was performed to examine the influence set by the individual study on the pooled ORs by deleting each study once in every genetic model. Consistently, the pooled estimate did not meet the statistical significance (S3 Fig).

### Publication bias

The funnel plot and Egger’s test were used to evaluate the publication bias between studies. The funnel plots for all six SNPs were symmetrical (S2 Fig). As a statistical method to test funnel plot symmetry, the Egger’s test did not detect significant publication bias in any SNP (rs6556547, *P* = 0.839; rs1816071, *P* = 0.693; rs1816072, *P* = 0.771; rs194072, *P* = 0.681; rs252944, *P* = 0.866; rs187269, *P* = 0.452).

## Discussion

The present meta-analysis study included a large sample size (51,491 patients and 74,667 controls) to investigate the potential association of 6 SNPs of *GABRB2* (rs6556547, rs1816071, rs1816072, rs194072, rs252944, and rs187269) with the occurrence of SCZ. The findings revealed no association. The ethnic subgroup analysis also showed no association in Asian and Caucasian populations.

Meta-analysis is an effective method to reanalyze multiple independent studies revolving around the same issue and obtain a general conclusion. The results of the present meta-analysis were inconsistent with those of two previous meta-analyses on *GABRB2* and SCZ. A possible reason might be the larger sample size used in the present study. Sampling error can be decreased by increasing the sample size of the study. The sample size of the previous two meta-analyses studies was limited. In the present meta-analysis, a total of 51,491 patients and 74,667 controls were included. Therefore, the sampling error was greatly reduced, making the assertion of the study more convincing. The weights carried by PGC samples in this meta-analysis were large. Moreover, the large sample size in GWAS might increase the statistical power to detect common SNP with a minor effect on the pathogenesis of SCZ.

Furthermore, the risk allele of rs1816072 with SCZ was different in Asian (C allele) and Caucasian (T allele) populations in the present study. These results were also detected in a previously published meta-analysis. Moreover, the minor allele frequency of rs6556547 was 0.178 in the East Asian population and 0.054 in the European population. It is suggested that subjects with different ethnicity exhibit genetic heterogeneity. Considering the ethnic heterogeneity, subgroup analyses were performed in Asian and Caucasian populations separately. In the present study, after combining data from GWASs in the Chinese Han population and PGC, the results of the subgroup analysis by ethnicity showed no association between *GABRB2* and SCZ.

The present meta-analysis still had a few limitations. First, the limited number of studies investigating the *GABRB2* SNPs implied the possibility of type II error [40]. Second, the different designs of the studies included in the meta-analysis might have influenced the association results, although the meta-regression analysis revealed no significant moderation by study design.

In conclusion, the present updated meta-analysis combined with GWAS Data of Han Chinese population and PGC suggested that *GABRB2* polymorphisms might not be associated with SCZ.

## Supporting information

S1 Fig Forest plot for meta-analysis on data of candidate gene association studies between *GABRB2* and schizophrenia. (A) Meta-analysis of rs6556547. (B) Meta-analysis of rs1816071. (C) Meta-analysis of rs1816072. (D) Meta-analysis of rs194072. (E) Meta-analysis of rs252944. (F) Meta-analysis of rs187269.
(DOCX)

S2 Fig Funnel plot for odds ratio of allele frequency comparison of SNPs in *GABRB2* combined with GWAS schizophrenia data. (A) Funnel plot of rs6556547. (B) Funnel plot of rs1816071. (C) Funnel plot of rs1816072. (D) Funnel plot of rs194072. (E) Funnel plot of rs252944. (F) Funnel plot of rs187269.
(DOCX)

S3 Fig Sensitivity analysis for SNPs in *GABRB2* combined with GWAS schizophrenia data. (A) rs6556547. (B) rs1816071. (C) rs1816072. (D) rs194072. (E) rs252944. (F) rs187269.
(DOCX)

S1 Table Meta-analysis on candidate gene association studies between *GABRB2* and schizophrenia in different ethnic subgroups without GWAS data. (DOCX)

S2 Table Meta-analytic results of *GABRB2* and schizophrenia in all populations combined with GWAS data. (DOCX)

S3 Table *P* value of Meta-regression results of different covariates. (DOCX)

S4 Table Summary of extracted data from the candidate gene association studies selected for the meta-analysis. (XLSX)

S1 File PRISMA checklist. (DOC)

S2 File PRISMA flow diagram. (DOC)

S3 File Meta-analysis-on-genetic-association-studies-form. (DOCX)

S4 File The list of excluded articles and the reasons for exclusion of each article. (DOCX)

## References

[1] Owen MJ, Sawa A, Mortensen PB. Schizophrenia. Lancet. 2016;388(10039):86–97. 10.1016/S0140-6736(15)01121-6 26777917 PMC4940219

[2] van Os J, Kenis G, Rutten BP. The environment and schizophrenia. Nature. 2010;468(7321):203–12. 10.1038/nature09563 21068828

[3] Tsuang MT, Stone WS, Faraone SV. Genes, environment and schizophrenia. Br J Psychiatry Suppl. 2001;40:s18–24. https://www.ncbi.nlm.nih.gov/pubmed/11315219 11315219 10.1192/bjp.178.40.s18

[4] Sullivan PF, Kendler KS, Neale MC. Schizophrenia as a complex trait: evidence from a meta-analysis of twin studies. Arch Gen Psychiatry. 2003;60(12):1187–92. 10.1001/archpsyc.60.12.1187 14662550

[5] Lewis DA, Hashimoto T, Volk DW. Cortical inhibitory neurons and schizophrenia. Nat Rev Neurosci. 2005;6(4):312–24. 10.1038/nrn1648 15803162

[6] Sieghart W, Fuchs K, Tretter V, Ebert V, Jechlinger M, Hoger H, et al Structure and subunit composition of GABA(A) receptors. Neurochem Int. 1999;34(5):379–85. https://www.ncbi.nlm.nih.gov/pubmed/10397365 10397365 10.1016/s0197-0186(99)00045-5

[7] Korpi ER, Sinkkonen ST. GABA(A) receptor subtypes as targets for neuropsychiatric drug development. Pharmacol Ther. 2006;109(1–2):12–32. 10.1016/j.pharmthera.2005.05.009 15996746

[8] Fatemi SH, Folsom TD, Rooney RJ, Thuras PD. Expression of GABAA alpha2-, beta1- and epsilon-receptors are altered significantly in the lateral cerebellum of subjects with schizophrenia, major depression and bipolar disorder. Transl Psychiatry. 2013;3:e303 10.1038/tp.2013.64 24022508 PMC3784760

[9] Han S, Tai C, Jones CJ, Scheuer T, Catterall WA. Enhancement of inhibitory neurotransmission by GABAA receptors having alpha2,3-subunits ameliorates behavioral deficits in a mouse model of autism. Neuron. 2014;81(6):1282–9. 10.1016/j.neuron.2014.01.016 24656250 PMC4079471

[10] Mizukami K, Grayson DR, Ikonomovic MD, Sheffield R, Armstrong DM. GABAA receptor beta 2 and beta 3 subunits mRNA in the hippocampal formation of aged human brain with Alzheimer-related neuropathology. Brain Res Mol Brain Res. 1998;56(1–2):268–72. https://www.ncbi.nlm.nih.gov/pubmed/9602147 9602147 10.1016/s0169-328x(97)00347-1

[11] Trudell JR, Messing RO, Mayfield J, Harris RA. Alcohol dependence: molecular and behavioral evidence. Trends Pharmacol Sci. 2014;35(7):317–23. 10.1016/j.tips.2014.04.009 24865944 PMC4089033

[12] Guidotti A, Auta J, Davis JM, Dong E, Grayson DR, Veldic M, et al GABAergic dysfunction in schizophrenia: new treatment strategies on the horizon. Psychopharmacology (Berl). 2005;180(2):191–205. 10.1007/s00213-005-2212-8 15864560

[13] Whiting PJ. The GABA-A receptor gene family: new targets for therapeutic intervention. Neurochem Int. 1999;34(5):387–90. https://www.ncbi.nlm.nih.gov/pubmed/10397366 10397366 10.1016/s0197-0186(99)00048-0

[14] Lewis CM, Levinson DF, Wise LH, DeLisi LE, Straub RE, Hovatta I, et al Genome scan meta-analysis of schizophrenia and bipolar disorder, part II: Schizophrenia. Am J Hum Genet. 2003;73(1):34–48. 10.1086/376549 12802786 PMC1180588

[15] Yamada K, Iwayama Y, Hattori E, Iwamoto K, Toyota T, Ohnishi T, et al Genome-wide association study of schizophrenia in Japanese population. PLoS One. 2011;6(6):e20468 10.1371/journal.pone.0020468 21674006 PMC3108953

[16] Cheng ZY, Wang XP, Schmid KL, Han XG. GABAB1 and GABAB2 receptor subunits co-expressed in cultured human RPE cells regulate intracellular Ca2+ via Gi/o-protein and phospholipase C pathways. Neuroscience. 2014;280:254–61. 10.1016/j.neuroscience.2014.09.021 25241062

[17] Zhao C, Xu Z, Chen J, Yu Z, Tong KL, Lo WS, et al Two isoforms of GABA(A) receptor beta2 subunit with different electrophysiological properties: Differential expression and genotypical correlations in schizophrenia. Mol Psychiatry. 2006;11(12):1092–105. 10.1038/sj.mp.4001899 16983389

[18] Mueller TM, Haroutunian V, Meador-Woodruff JH. N-Glycosylation of GABAA receptor subunits is altered in Schizophrenia. Neuropsychopharmacology. 2014;39(3):528–37. 10.1038/npp.2013.190 23917429 PMC3895232

[19] Zong L, Zhou L, Hou Y, Zhang L, Jiang W, Zhang W, et al Genetic and epigenetic regulation on the transcription of GABRB2: Genotype-dependent hydroxymethylation and methylation alterations in schizophrenia. J Psychiatr Res. 2017;88:9–17. 10.1016/j.jpsychires.2016.12.019 28063323

[20] Zhao X, Qin SY, Shi YY, Zhang AP, Zhang J, Bian L, et al Systematic study of association of four GABAergic genes: Glutamic acid decarboxylase 1 gene, glutamic acid decarboxylase 2 gene, GABA(B) receptor 1 gene and GABA(A) receptor subunit beta 2 gene, with schizophrenia using a universal DNA microarray. Schizophrenia Research. 2007;93(1–3):374–84. 10.1016/j.schres.2007.02.023 WOS:000247663200043 17412563

[21] Lo WS, Harano M, Gawlik M, Yu Z, Chen J, Pun FW, et al GABRB2 association with schizophrenia: commonalities and differences between ethnic groups and clinical subtypes. Biol Psychiatry. 2007;61(5):653–60. 10.1016/j.biopsych.2006.05.003 16950232

[22] Lo WS, Lau CF, Xuan Z, Chan CF, Feng GY, He L, et al Association of SNPs and haplotypes in GABAA receptor beta2 gene with schizophrenia. Mol Psychiatry. 2004;9(6):603–8. 10.1038/sj.mp.4001461 14699426

[23] Petryshen TL, Middleton FA, Tahl AR, Rockwell GN, Purcell S, Aldinger KA, et al Genetic investigation of chromosome 5q GABAA receptor subunit genes in schizophrenia. Mol Psychiatry. 2005;10(12):1074–88, 57. 10.1038/sj.mp.4001739 16172613

[24] Liu JX, Shi YY, Tang W, Guo TW, Li DW, Yang YF, et al Positive association of the human GABA-A-receptor beta 2 subunit gene haplotype with schizophrenia in the Chinese Han population. Biochem Bioph Res Co. 2005;334(3):817–23. 10.1016/j.bbrc.2005.06.167 WOS:000231226300012 16023997

[25] Ikeda M, Iwata N, Suzuki T, Kitajima T, Yamanouchi Y, Kinoshita Y, et al Association analysis of chromosome 5 GABAA receptor cluster in Japanese schizophrenia patients. Biol Psychiatry. 2005;58(6):440–5. 10.1016/j.biopsych.2005.05.002 15993854

[26] Jamra RA, Becker T, Klopp N, Dahdouh F, Schulze TG, Gross M, et al No evidence for an association between variants at the gamma-amino-n-butyric acid type A receptor beta2 locus and schizophrenia. Psychiatr Genet. 2007;17(1):43–5. 10.1097/YPG.0b013e32801118cd 17167345

[27] Allen NC, Bagade S, McQueen MB, Ioannidis JP, Kavvoura FK, Khoury MJ, et al Systematic meta-analyses and field synopsis of genetic association studies in schizophrenia: the SzGene database. Nat Genet. 2008;40(7):827–34. 10.1038/ng.171 18583979

[28] Shi J, Gershon ES, Liu C. Genetic associations with schizophrenia: meta-analyses of 12 candidate genes. Schizophr Res. 2008;104(1–3):96–107. 10.1016/j.schres.2008.06.016 18715757 PMC2562556

[29] Pun FW, Zhao C, Lo WS, Ng SK, Tsang SY, Nimgaonkar V, et al Imprinting in the schizophrenia candidate gene GABRB2 encoding GABA(A) receptor beta(2) subunit. Mol Psychiatr. 2011;16(5):557–68. 10.1038/mp.2010.47 WOS:000289884400010 20404824

[30] Tsang SY, Zhong S, Mei L, Chen J, Ng SK, Pun FW, et al Social cognitive role of schizophrenia candidate gene GABRB2. PLoS One. 2013;8(4):e62322 10.1371/journal.pone.0062322 23638040 PMC3634734

[31] Balan S, Yamada K, Iwayama Y, Hashimoto T, Toyota T, Shimamoto C, et al Comprehensive association analysis of 27 genes from the GABAergic system in Japanese individuals affected with schizophrenia. Schizophr Res. 2017;185:33–40. 10.1016/j.schres.2017.01.003 28073605

[32] Manolio TA. Genomewide association studies and assessment of the risk of disease. N Engl J Med. 2010;363(2):166–76. 10.1056/NEJMra0905980 20647212

[33] Schizophrenia Psychiatric Genome-Wide Association Study C. Genome-wide association study identifies five new schizophrenia loci. Nat Genet. 2011;43(10):969–76. 10.1038/ng.940 21926974 PMC3303194

[34] Ripke S, O'Dushlaine C, Chambert K, Moran JL, Kahler AK, Akterin S, et al Genome-wide association analysis identifies 13 new risk loci for schizophrenia. Nat Genet. 2013;45(10):1150–9. 10.1038/ng.2742 23974872 PMC3827979

[35] Schizophrenia Working Group of the Psychiatric Genomics C. Biological insights from 108 schizophrenia-associated genetic loci. Nature. 2014;511(7510):421–7. 10.1038/nature13595 25056061 PMC4112379

[36] Yu H, Yan H, Li J, Li Z, Zhang X, Ma Y, et al Common variants on 2p16.1, 6p22.1 and 10q24.32 are associated with schizophrenia in Han Chinese population. Mol Psychiatry. 2017;22(7):954–60. 10.1038/mp.2016.212 27922604

[37] Li Z, Chen J, Yu H, He L, Xu Y, Zhang D, et al Genome-wide association analysis identifies 30 new susceptibility loci for SCZ. Nat Genet. 2017;49(11):1576–83. 10.1038/ng.3973 28991256

[38] Kazeem GR, Farrall M. Integrating case-control and TDT studies. Ann Hum Genet. 2005;69(Pt 3):329–35. 10.1046/j.1529-8817.2005.00156.x 15845037

[39] Lohmueller KE, Pearce CL, Pike M, Lander ES, Hirschhorn JN. Meta-analysis of genetic association studies supports a contribution of common variants to susceptibility to common disease. Nat Genet. 2003;33(2):177–82. 10.1038/ng1071 12524541

[40] Dudbridge F, Gusnanto A. Estimation of significance thresholds for genomewide association scans. Genet Epidemiol. 2008;32(3):227–34. 10.1002/gepi.20297 18300295 PMC2573032

