# Supplementary material for: Meta-analysis of GABRB2 polymorphisms and the risk of schizophrenia combined with GWAS data of the Han Chinese population and psychiatric genomics consortium
Source: PLoS One. 2018 Jun 12;13(6):e0198690. doi: 10.1371/journal.pone.0198690 (PMC5997335; doi:10.1371/journal.pone.0198690)
Supplement: S1 Fig — (A) Meta-analysis of rs6556547. (B) Meta-analysis of rs1816071. (C) Meta-analysis of rs1816072. (D) Meta-analysis of rs194072. (E) Meta-analysis of rs252944. (F) Meta-analysis of rs187269. (DOCX) [file pone.0198690.s001.docx]

**S1 Fig. Forest plot for meta-analysis on data of candidate gene association studies between *GABRB2* and schizophrenia.**

**A. Meta-analysis of rs6556547**

**
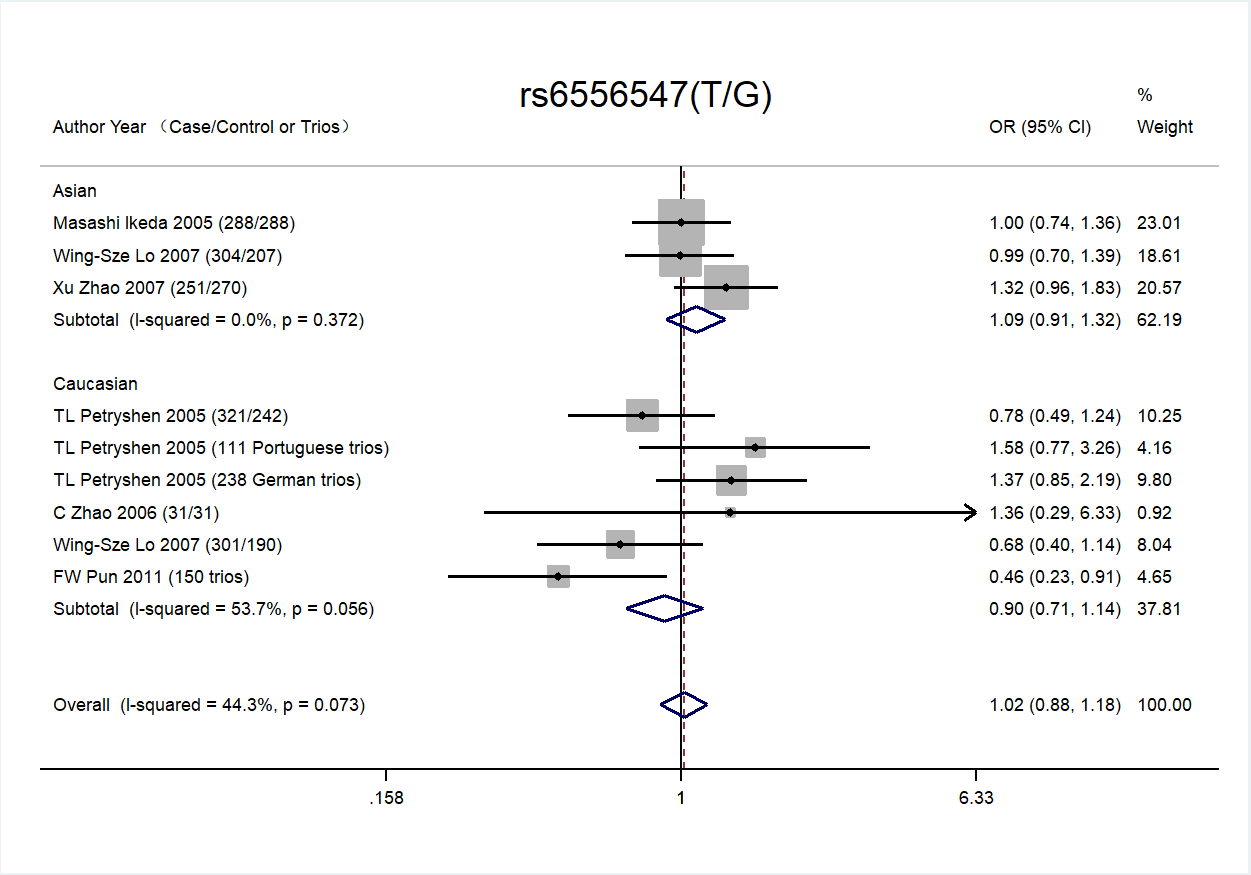
**

1. **Meta-analysis of rs1816071**

**
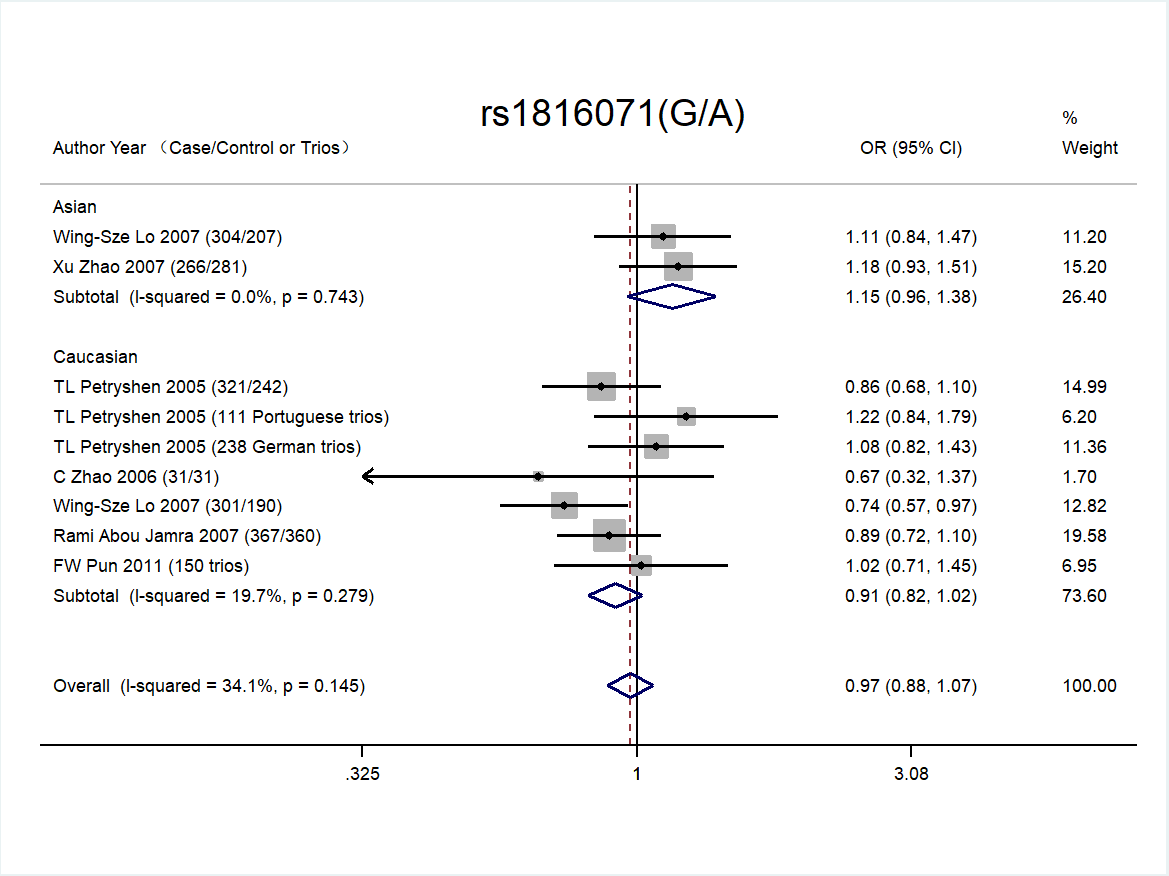
**

**C. Meta-analysis of rs1816072**

**
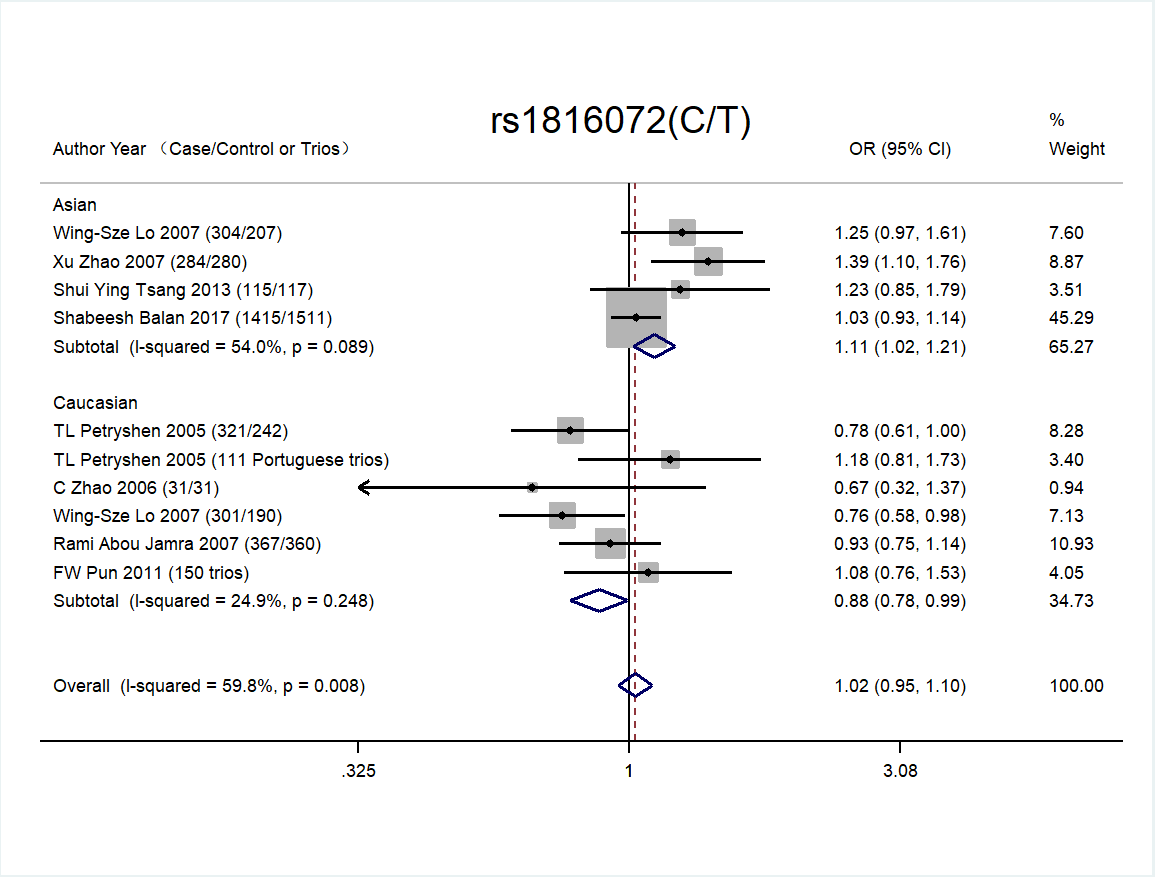
**

**D. Meta-analysis of rs194072**

**
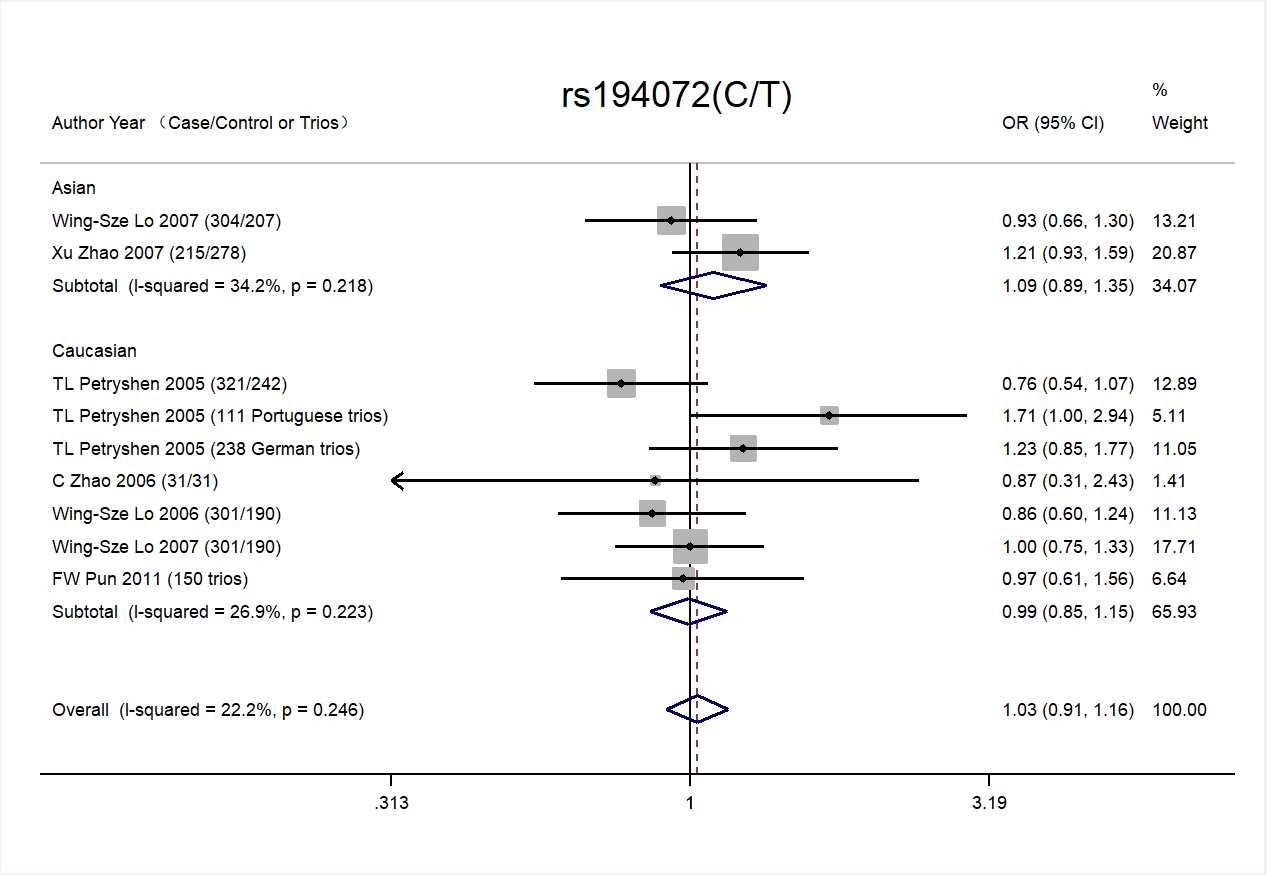
**

**E. Meta-analysis of rs252944**

**
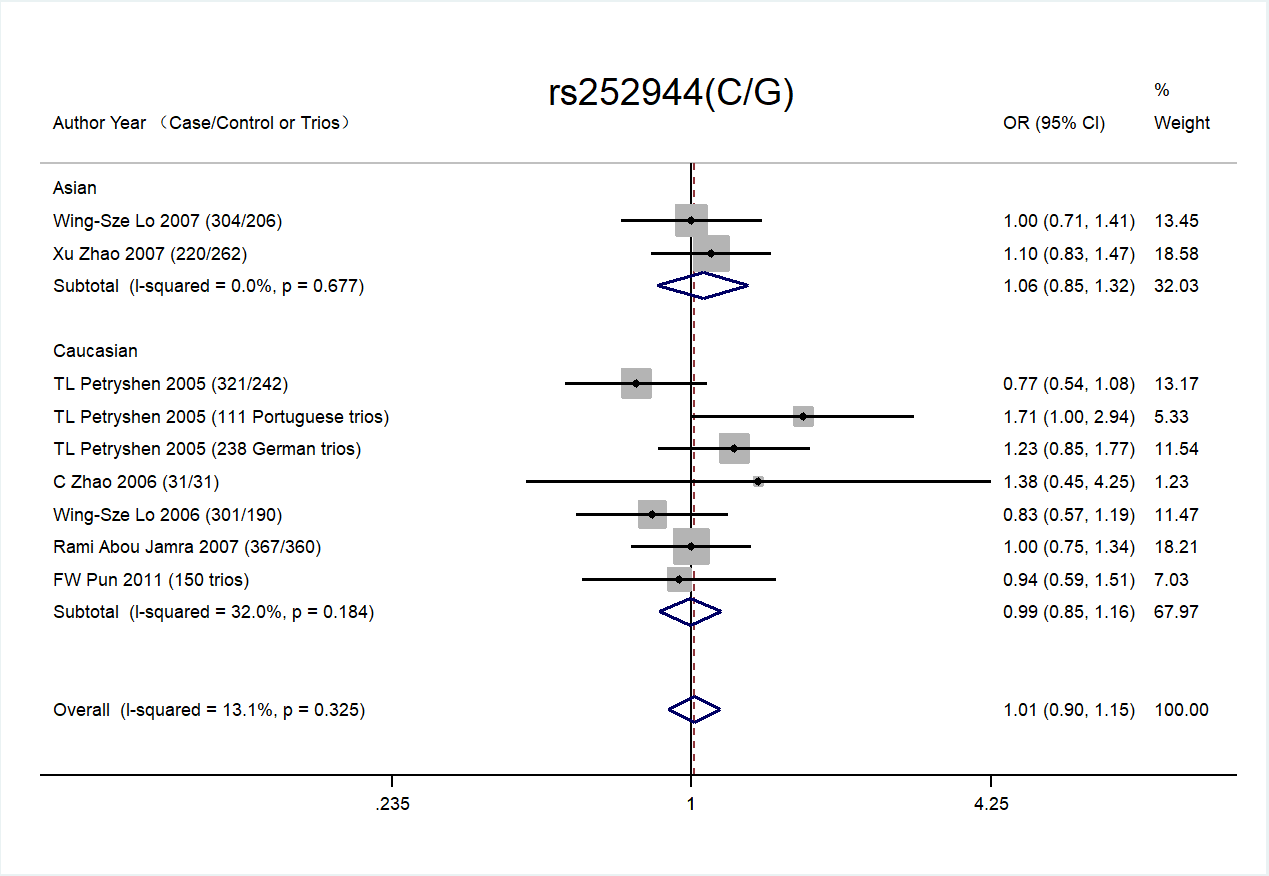
**

**F. Meta-analysis of rs187269**

**
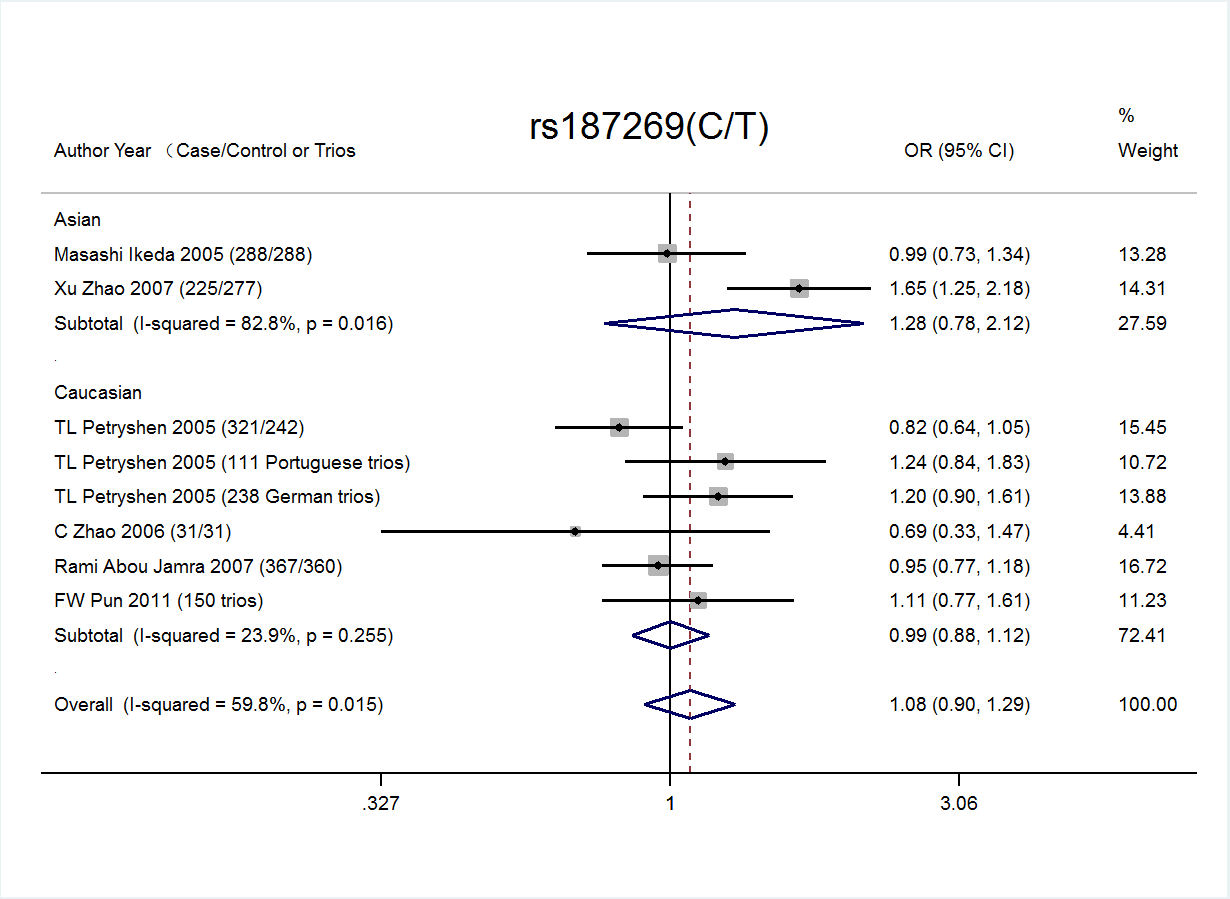
**
