# Supplementary material for: Meta-analysis of GABRB2 polymorphisms and the risk of schizophrenia combined with GWAS data of the Han Chinese population and psychiatric genomics consortium
Source: PLoS One. 2018 Jun 12;13(6):e0198690. doi: 10.1371/journal.pone.0198690 (PMC5997335; doi:10.1371/journal.pone.0198690)
Supplement: S2 Fig — (A) Funnel plot of rs6556547. (B) Funnel plot of rs1816071. (C) Funnel plot of rs1816072. (D) Funnel plot of rs194072. (E) Funnel plot of rs252944. (F) Funnel plot of rs187269. (DOCX) [file pone.0198690.s002.docx]

**S2 Fig. Funnel plot for odds ratio of allele frequency comparison of SNPs in *GABRB2* combined with GWAS schizophrenia data.**

**A. Funnel plot of rs6556547**

**B. Funnel plot of rs1816071**

**C. Funnel plot of rs1816072**

**D. Funnel plot of rs194072**

**E. Funnel plot of rs252944**

**F. Funnel plot of rs187269**
