# Supplementary material for: Meta-analysis of GABRB2 polymorphisms and the risk of schizophrenia combined with GWAS data of the Han Chinese population and psychiatric genomics consortium
Source: PLoS One. 2018 Jun 12;13(6):e0198690. doi: 10.1371/journal.pone.0198690 (PMC5997335; doi:10.1371/journal.pone.0198690)
Supplement: S3 Fig — (A) rs6556547. (B) rs1816071. (C) rs1816072. (D) rs194072. (E) rs252944. (F) rs187269. (DOCX) [file pone.0198690.s003.docx]

**S3 Fig. Sensitivity analysis for SNPs in *GABRB2* combined with GWAS schizophrenia data.**

1. **rs6556547**

1. **rs1816071**

1. **rs1816072**

1. **rs194072**

1. **rs252944**

1. **rs187269**
