# Supplementary material for: Meta-analysis of GABRB2 polymorphisms and the risk of schizophrenia combined with GWAS data of the Han Chinese population and psychiatric genomics consortium
Source: PLoS One. 2018 Jun 12;13(6):e0198690. doi: 10.1371/journal.pone.0198690 (PMC5997335; doi:10.1371/journal.pone.0198690)
Supplement: S1 Table — (DOCX) [file pone.0198690.s004.docx]

**S1 Table. Meta-analysis on candidate gene association studies between *GABRB2* and schizophrenia in different ethnic subgroups without GWAS data.**

| SNP | Population | OR | CI （95%） | *Z* | *P* | Heterogeneity | | |
| --- | --- | --- | --- | --- | --- | --- | --- | --- |
|  |  |  |  |  |  | Q | *P* | I-squared |
| rs6556547 | Asian | 1.09 | 0.91-1.32 | 0.93 | 0.350 | 1.98 | 0.372 | 0.00% |
|  | Caucasian | 0.90 | 0.62-1.31 | 0.54 | 0.588 | 10.80 | 0.056 | 53.70% |
|  | overall | 1.02 | 0.88-1.18 | 0.21 | 0.837 | 14.35 | 0.073 | 44.30% |
| rs1816071 | Asian | 1.15 | 0.96-1.39 | 1.53 | 0.127 | 0.11 | 0.743 | 0.00% |
|  | Caucasian | 0.91 | 0.82-1.02 | 1.61 | 0.108 | 7.48 | 0.279 | 19.70% |
|  | overall | 0.97 | 0.89-1.07 | 0.59 | 0.553 | 12.14 | 0.145 | 34.10% |
| rs1816072 | Asian | 1.18 | 1.01-1.39 | 2.02 | **0.043** | 6.52 | 0.089 | 54.00% |
|  | Caucasian | 0.88 | 0.78-0.99 | 2.07 | **0.038** | 6.65 | 0.248 | 24.90% |
|  | overall | 1.02 | 0.90-1.16 | 0.34 | 0.731 | 22.39 | 0.008 | 59.80% |
| rs194072 | Asian | 1.10 | 0.89-1.35 | 0.85 | 0.396 | 1.52 | 0.218 | 34.20% |
|  | Caucasian | 0.99 | 0.86-1.15 | 0.09 | 0.928 | 8.21 | 0.223 | 26.90% |
|  | overall | 1.03 | 0.91-1.16 | 0.42 | 0.673 | 10.28 | 0.246 | 22.20% |
| rs252944 | Asian | 1.06 | 0.85-1.32 | 0.5 | 0.615 | 0.17 | 0.677 | 0.00% |
|  | Caucasian | 0.99 | 0.86-1.16 | 0.08 | 0.934 | 8.82 | 0.184 | 32.00% |
|  | overall | 1.01 | 0.90-1.15 | 0.22 | 0.829 | 9.20 | 0.325 | 13.10% |
| rs187269 | Asian | 1.28 | 0.78-2.12 | 0.97 | 0.333 | 5.82 | 0.016 | 82.80% |
|  | Caucasian | 0.99 | 0.88-1.12 | 0.11 | 0.911 | 6.57 | 0.255 | 23.90% |
|  | overall | 1.08 | 0.90-1.29 | 0.85 | 0.398 | 17.41 | 0.015 | 59.80% |

Abbreviations: OR, odds ratio; CI, confidence intervals. *P* value in bold font represents statistical significance.
