# Supplementary material for: Meta-analysis of GABRB2 polymorphisms and the risk of schizophrenia combined with GWAS data of the Han Chinese population and psychiatric genomics consortium
Source: PLoS One. 2018 Jun 12;13(6):e0198690. doi: 10.1371/journal.pone.0198690 (PMC5997335; doi:10.1371/journal.pone.0198690)
Supplement: S2 Table — (DOCX) [file pone.0198690.s005.docx]

**S2 Table. Meta-analytic results of *GABRB2* and schizophrenia in all populations combined with GWAS data.**

| SNP | Population | OR | CI （95%） | *Z* | *P* | Heterogeneity | | |
| --- | --- | --- | --- | --- | --- | --- | --- | --- |
|  |  |  |  |  |  | Q | *P* | I-squared |
| rs6556547 | overall | 1.00 | 0.97-1.03 | 0.22 | 0.827 | 17.10 | 0.105 | 35.7% |
| rs1816071 | overall | 1.00 | 0.99-1.02 | 0.39 | 0.697 | 13.73 | 0.248 | 19.9% |
| rs1816072 | overall | 1.00 | 0.98-1.02 | 0.10 | 0.919 | 22.91 | 0.028 | 47.6% |
| rs194072 | overall | 1.00 | 0.97-1.02 | 0.41 | 0.678 | 14.24 | 0.220 | 22.8% |
| rs252944 | overall | 1.01 | 0.99-1.04 | 0.79 | 0.429 | 12.61 | 0.320 | 12.8% |
| rs187269 | overall | 1.00 | 0.98-1.02 | 0.06 | 0.954 | 19.14 | 0.038 | 47.8% |

Abbreviations: OR, odds ratio; CI, confidence intervals.
