# Supplementary material for: Meta-analysis of GABRB2 polymorphisms and the risk of schizophrenia combined with GWAS data of the Han Chinese population and psychiatric genomics consortium
Source: PLoS One. 2018 Jun 12;13(6):e0198690. doi: 10.1371/journal.pone.0198690 (PMC5997335; doi:10.1371/journal.pone.0198690)
Supplement: S3 Table — (DOCX) [file pone.0198690.s006.docx]

**S3 Table. *P* value of Meta-regression results of different covariates.**

| SNP | Sample size | Year of publication | Study design |
| --- | --- | --- | --- |
| rs6556547 | 0.818 | 0.631 | 0.907 |
| rs1816071 | 0.720 | 0.371 | 0.160 |
| rs1816072 | 0.875 | 0.823 | 0.958 |
| rs194072 | 0.815 | 0.742 | 0.305 |
| rs252944 | 0.811 | 0.785 | 0.202 |
| rs187269 | 0.673 | 0.614 | 0.670 |
