# Supplementary material for: Meta-analysis of GABRB2 polymorphisms and the risk of schizophrenia combined with GWAS data of the Han Chinese population and psychiatric genomics consortium
Source: PLoS One. 2018 Jun 12;13(6):e0198690. doi: 10.1371/journal.pone.0198690 (PMC5997335; doi:10.1371/journal.pone.0198690)
Supplement: S2 File — (DOC) [file pone.0198690.s009.doc]

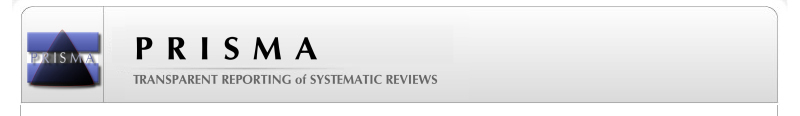
**PRISMA 2009 Flow Diagram**

**Screening**

**Included**

**Eligibility**

**Identification**

Records identified through database searching
(n = 113 )

Additional records identified through other sources
(n = 11 )

Records after duplicates removed
(n = 82 )

Records screened
(n = 82 )

Records excluded
(n = 54 )

Full-text articles assessed for eligibility
(n = 28 )

Full-text articles excluded, with reasons
(n = 16 )

Studies included in qualitative synthesis
(n = 12 )

Studies included in quantitative synthesis (meta-analysis)
(n = 12 )
