# Supplementary material for: Meta-analysis of GABRB2 polymorphisms and the risk of schizophrenia combined with GWAS data of the Han Chinese population and psychiatric genomics consortium
Source: PLoS One. 2018 Jun 12;13(6):e0198690. doi: 10.1371/journal.pone.0198690 (PMC5997335; doi:10.1371/journal.pone.0198690)
Supplement: S4 File — (DOCX) [file pone.0198690.s011.docx]

After reading full-text studies, 16 studies were excluded with reasons

**（1） 2 review studies**

1. Moalic JM, Le Strat Y, Lepagnol-Bestel AM, Ramoz N, Loe-Mie Y, Maussion G, et al. Primate-accelerated evolutionary genes: novel routes to drug discovery in psychiatric disorders. Current medicinal chemistry. 2010;17(13):1300-16. PMID: 20166940

2. Cherlyn SY, Woon PS, Liu JJ, Ong WY, Tsai GC, Sim K. Genetic association studies of glutamate, GABA and related genes in schizophrenia and bipolar disorder: a decade of advance. Neuroscience and biobehavioral reviews. 2010;34(6):958-77. http://dx.doi.org/10.1016/j.neubiorev.2010.01.002 PMID: 20060416

**（2） 2 animal studies**

1. Wang L, Jiang W, Lin Q, Zhang Y, Zhao C. DNA methylation regulates gabrb2 mRNA expression: developmental variations and disruptions in l-methionine-induced zebrafish with schizophrenia-like symptoms. Genes, brain, and behavior. 2016;15(8):702-10. http://dx.doi.org/10.1111/gbb.12315 PMID: 27509263

2. Pan B, Lian J, Huang XF, Deng C. Aripiprazole Increases the PKA Signalling and Expression of the GABAA Receptor and CREB1 in the Nucleus Accumbens of Rats. Journal of molecular neuroscience : MN. 2016;59(1):36-47. http://dx.doi.org/10.1007/s12031-016-0730-y PMID: 26894264

**（3） 3 studies that has duplicated participants with others**

1. Liu J, Shi Y, Tang W, Guo T, Li D, Yang Y, et al. Positive association of the human GABA-A-receptor beta 2 subunit gene haplotype with schizophrenia in the Chinese Han population. Biochem Biophys Res Commun. 2005;334(3):817-23. http://dx.doi.org/10.1016/j.bbrc.2005.06.167 PMID: 16023997

2. Lo WS, Lau CF, Xuan Z, Chan CF, Feng GY, He L, et al. Association of SNPs and haplotypes in GABAA receptor beta2 gene with schizophrenia. Mol Psychiatry. 2004;9(6):603-8. http://dx.doi.org/10.1038/sj.mp.4001461 PMID: 14699426

3. Yu Z, Chen J, Shi H, Stoeber G, Tsang SY, Xue H. Analysis of GABRB2 association with schizophrenia in German population with DNA sequencing and one-label extension method for SNP genotyping. Clinical biochemistry. 2006;39(3):210-8. http://dx.doi.org/10.1016/j.clinbiochem.2006.01.009 PMID: 16472798

**（4） 1 studies with no complete data[8]**

1. Lo WS, Xu Z, Yu Z, Pun FW, Ng SK, Chen J, et al. Positive selection within the Schizophrenia-associated GABA(A) receptor beta(2) gene. PLoS One. 2007;2(5):e462. http://dx.doi.org/10.1371/journal.pone.0000462 PMID: 17520021

**（5）7 studies that did not explore the polymorphisms**

1. Mueller TM, Remedies CE, Haroutunian V, Meador-Woodruff JH. Abnormal subcellular localization of GABAA receptor subunits in schizophrenia brain. Transl Psychiatry. 2015;5:e612. http://dx.doi.org/10.1038/tp.2015.102 PMID: 26241350

2. Lindberg PG, Teremetz M, Charron S, Kebir O, Saby A, Bendjemaa N, et al. Altered cortical processing of motor inhibition in schizophrenia. Cortex; a journal devoted to the study of the nervous system and behavior. 2016;85:1-12. http://dx.doi.org/10.1016/j.cortex.2016.09.019 PMID: 27770667

3. Zhao C, Xu Z, Wang F, Chen J, Ng SK, Wong PW, et al. Alternative-splicing in the exon-10 region of GABA(A) receptor beta(2) subunit gene: relationships between novel isoforms and psychotic disorders. PLoS One. 2009;4(9):e6977. http://dx.doi.org/10.1371/journal.pone.0006977 PMID: 19763268

4. Zhao C, Wang F, Pun FW, Mei L, Ren L, Yu Z, et al. Epigenetic regulation on GABRB2 isoforms expression: developmental variations and disruptions in psychotic disorders. Schizophr Res. 2012;134(2-3):260-6. http://dx.doi.org/10.1016/j.schres.2011.11.029 PMID: 22206711

5. Chen J, Tsang SY, Zhao CY, Pun FW, Yu Z, Mei L, et al. GABRB2 in schizophrenia and bipolar disorder: disease association, gene expression and clinical correlations. Biochemical Society transactions. 2009;37(Pt 6):1415-8. http://dx.doi.org/10.1042/bst0371415 PMID: 19909288

6. Beneyto M, Abbott A, Hashimoto T, Lewis DA. Lamina-specific alterations in cortical GABA(A) receptor subunit expression in schizophrenia. Cerebral cortex (New York, NY : 1991). 2011;21(5):999-1011. http://dx.doi.org/10.1093/cercor/bhq169 PMID: 20843900

7. Ng SK, Lo WS, Pun FW, Zhao C, Yu Z, Chen J, et al. A recombination hotspot in a schizophrenia-associated region of GABRB2. PLoS One. 2010;5(3):e9547. http://dx.doi.org/10.1371/journal.pone.0009547 PMID: 20221451

**（8） 1 study that did not investigate the polymorphisms included in the present meta-analysis**

1. Zong L, Zhou L, Hou Y, Zhang L, Jiang W, Zhang W, et al. Genetic and epigenetic regulation on the transcription of GABRB2: Genotype-dependent hydroxymethylation and methylation alterations in schizophrenia. J Psychiatr Res. 2017;88:9-17. http://dx.doi.org/10.1016/j.jpsychires.2016.12.019 PMID: 28063323
